# Investigation of co-existing bacteria in platelets by employing long-term culturomics and metagenomics

**DOI:** 10.3389/fcimb.2025.1607554

**Published:** 2025-09-10

**Authors:** Qiqi Wang, Mengyi Zhao, Anqing Liu, Yuwei Zhao, Zhenxin Fan, Yang Huang, Zhan Gao, Miao He

**Affiliations:** ^1^ Institute of Blood Transfusion, Chinese Academy of Medical Sciences, Chengdu, China; ^2^ Department of Laboratory Medicine, Chengdu Fifth People’s Hospital, Chengdu, Sichuan, China; ^3^ Chengdu Blood Center, Chengdu, China; ^4^ Key Laboratory of Bioresources and Ecoenvironment, Ministry of Education, College of Life Sciences, Sichuan University, Chengdu, China

**Keywords:** platelets, metagenomics, culturomics, bacteria, healthy bacteremia

## Abstract

**Introduction:**

Bacterial contamination of platelets presents a substantial risk in transfusion medicine. Conventional detection approaches have limitations in sensitivity and bacterial coverage. In this study we employed culturomics and metagenomics to investigate co-existent bacteria in platelets, aiming to enhance transfusion safety and explore healthy bacteremia.

**Methods:**

Platelet from 6 healthy donors underwent a 30-days extensive cultivation and isolation procedure using in-house culturomics.

**Results:**

16S rRNA sequencing identified 90 bacterial strains across 3 phyla, 5 classes, 5orders, 7 families, 9 genera, and 23 species. Metagenomics sequencing revealed greater microbial diversity, detecting an average of 3018 microbial species per sample. The bacteria concurrently detected by both culturomics and metagenomics included species from Firmicutes, Actinobacteria, and Proteobacteria.

**Discussion:**

This combined approach validates the presence of bacteria in platelets, likely originating from the skin, gut, oral cavity, environment, or bloodstream, providing a comprehensive strategy for bacterial identification in transfusion products.

## 1 Introduction

Bacterial contamination represents a critical concern in transfusion medicine. The primary sources of blood component contamination include skin microbiota (Levy et al., 2018) from donors, environmental contamination (Horth et al., 2018) during collection, inadequate hand hygiene of collection personnel (Levy et al., 2018), and recent infections in donors. Platelets, stored under constant agitation at room temperature (22 ± 2°C), provide an ideal environment for bacterial proliferation, allowing low bacterial concentrations (<1 CFU/mL) to rapidly grow to high levels (>10^8^ CFU/mL) (Stormer and Vollmer, 2014). This significantly increases the risk of bacterial contamination in platelet products. Platelet bacterial contamination can precipitate severe transfusion-related adverse reactions, with clinical relevance confirmed through hemovigilance reports (Greco et al., 2010). The reported complications span from mild febrile sepsis reactions to severe septic shock, which may result in patient mortality (Horth et al., 2018; Dumont et al., 2010). Furthermore, transfusion-transmitted bacteremia and subsequent delayed infections impose additional challenges on clinical management. Consequently, the implementation of stringent quality control measures to mitigate the risk of transfusion-transmitted infectious diseases (TTIDs) is of paramount importance (Jacobs et al., 2008; Benjamin et al., 2017).

At present, a variety of strategies are being utilized to reduce bacterial contamination in platelets, including strict donor selection, appropriate skin preparation at the venipuncture site, effective skin disinfection, diversion of the initial portion of collected blood, leukocyte reduction, and adherence to good manufacturing practices (GMP) (Walther-Wenke et al., 2011). Although these measures have significantly reduced contamination rates, they are incapable of completely eradicating the risk. Research findings suggest that even under strict operational protocols, a certain percentage of platelets still end up being contaminated. In recent years, several transfusion-related adverse reactions caused by bacterial contamination of platelets have been documented in Europe and North America (Thyer et al., 2018; Mcdonald et al., 2017). However, such incidents are seldom reported in China, potentially attributable to several factors: 1) platelet bacterial contamination screening has not yet become a routine procedure in Chinese blood centers; and 2) the number of platelet donors in China is relatively small, with a blood donation rate of merely 12 per thousand population (National Health Commission of the People’s Republic of China, 2019). This insufficient donor pool gives rise to clinical blood shortages, which subsequently exert pressure on blood collection and supply. Consequently, there is frequently insufficient time to perform routine bacterial cultures for each platelet unit. Ordinarily, only about four platelet units per month (National Health Commission of the People’s Republic of China, 2022) are sampled and tested for bacterial contamination, while the remaining units are released directly for clinical use without undergoing testing. The potential pathogens in inadequately screened platelet products may not cause disease in healthy individuals but pose a significant risk of infection to immunocompromised recipients (Vollmer et al., 2014; Shander et al., 2016).

As bacterial culture remains one of the most effective and essential methods for early detection of contamination during platelet storage, studies have shown that bacterial culture can reduce septic transfusion reactions (STRs) by 50%-75% (Mcdonald, 2011). Currently, the majority of blood centers in China rely on fully automated bacterial culture systems to detect bacterial contamination in platelets (Bei et al., 2018). However, these systems exhibit conspicuous limitations, including a high false-negative rate and a restricted range of detectable bacterial species (Lagier et al., 2016; Nussbaumer et al., 2007). Consequently, refining culture techniques to boost the detection of bacteria in platelets is an immediate and pressing priority. Microbial high-throughput culturomics is a technique that makes use of multiple culture conditions to facilitate the cultivation and screening of a broad spectrum of bacteria, even those that are uncultivable under standard conditions (Seng et al., 2013). This approach has been widely applied to the research on the human gut microbiota (Lagier et al., 2012), skin (Timm et al., 2020), vagina (Wolf et al., 2021), oral cavity, and other body sites. The development of culturomics has reshaped the traditional understanding of “uncultivable” microbes and demonstrated significant potential for isolating fastidious bacteria.

Meanwhile, With the progress of sequencing technologies and bioinformatics, researchers have embarked on studying microbial communities at the genetic level. Metagenomics next-generation sequencing (mNGS) empowers the detection of all microbial communities within a specific niche without the necessity for isolation, cultivation, or amplification steps. This approach effectively mitigates the biases inherent in traditional culture-based and PCR-based methods, allowing for more comprehensive and accurate microbial profiling (Lakshmanan and Liu, 2025; Knight et al., 2018; La Reau et al., 2023). mNGS has now been utilized to investigate novel and re-emerging transfusion-related pathogens. It has successfully identified various common pathogens (Brito et al., 2018; Gao et al., 2020) and opportunistic pathogens (Cristina et al., 2019; Laupland et al., 2022) in samples sourced from healthy voluntary blood donors. Furthermore, studies have demonstrated that mNGS, when applied to the analysis of plasma from healthy individuals, successfully detects a wide range of bacteria, parasites, and non-pathogenic viruses (Xu et al., 2018). While mNGS has revealed the rich diversity of microbial communities, it has also brought to light the fact that numerous bacteria remain uncultivated (Lagier et al., 2018).

In light of these findings, this study combines microbial high-throughput culturomics and mNGS to comprehensively detect the microbes in qualified donations in order to investigate the potential pathogens that may pose a threat to blood safety. By integrating these complementary approaches, this study aims to overcome the limitations of current detection methods, investigate the composition of cultivable bacteria in platelets, and furnish a scientific foundation for enhancing the blood safety.

## 2 Materials and methods

### 2.1 Sample collection and processing

Single-donor apheresis platelet samples were collected from 6 healthy voluntary blood donors at the Chengdu Blood Center between May 2022 and November 2023, comprising a total of 12 therapeutic units. The study was approved by the Ethics Review Committee of the Institute of Blood Transfusion, Chinese Academy of Medical Sciences (Ethics Approval Number: 2021011). For analysis, we defined one analytical sample per donor (n = 6). From each analytical sample, 10 mL was allocated to metagenomic sequencing and 100 mL to long-term culturomics. Processing was conducted in three laboratory batches (two analytical samples per batch), with one PBS-only culture control included in each batch and handled in parallel using the same culture and day-30 metagenomic workflow.

Collections and sampling followed the blood center’s routine, industry-standard antisepsis and sterility procedures. Phlebotomy sites were prepared using 2% chlorhexidine gluconate in 70% isopropyl alcohol, with a friction scrub for 30–60 s covering an area >5–7 cm in diameter, followed by 30 s of air-drying; no-touch venipuncture was performed, and the site was re-prepped if repalpated. For donors with chlorhexidine sensitivity, 10% povidone–iodine (alcohol-based two-step) was used as an alternative per institutional policy. We diverted the initial 20–30 mL of blood into an integrated first-diversion pouch before the main collection. All sampling was performed via sterile ports using new sterile needles/syringes, after wiping the port membrane with 70% isopropyl alcohol and allowing it to dry. Sample aliquots were processed in a biological safety cabinet with dedicated, pre-cleaned instruments.

### 2.2 Microbiological culturomics analysis

#### 2.2.1 Culture media and composition

The study used various media (Diakite et al., 2020) including COS liquid medium (Cos), YCFA medium (YCFA), Christensenella medium (Chr), Schaedler medium (Sch), R-Medium (R), R-Medium supplemented with lamb serum (R+), CNA solid medium (CNS), 2216E medium (2216E), Columbia blood agar plates (blood plates), anaerobic blood culture bottles (BPN, Catalog No: 259790, Mérieux, France), aerobic blood culture bottles (BPA, Catalog No: 259789, Mérieux, France), and additional combinations (BPN+B, BPN+L, BPN+B+L). Details of the medium compositions are provided in **Supplementary Table**.

#### 2.2.2 Culture conditions

**Figure 1:**
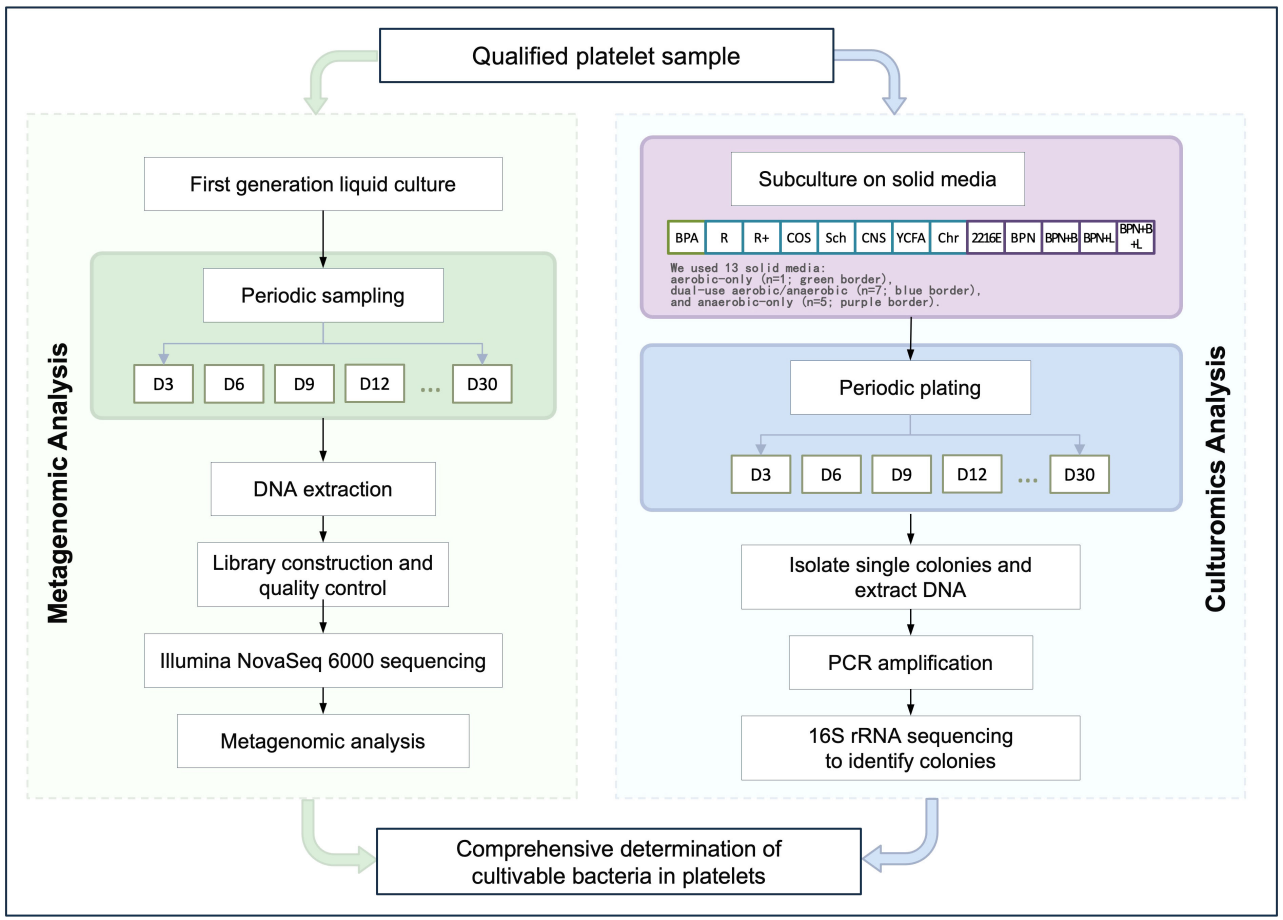
Workflow of Platelet Culturomics and Metagenomics.

Six platelet samples were inoculated into liquid media and cultured under both aerobic and anaerobic conditions. For aerobic culture, each 5 mL sample was inoculated into eight types of liquid media (R, R+, Cos, Sch, CNS, YCFA, Chr, BPA) and incubated at 37°C for 24 hours. A 100 µL aliquot was streaked onto 5% sheep blood agar plates and further incubated, with this process repeated every three days for a total of 30 days. For anaerobic culture, each 5 mL sample was inoculated into 12 types of liquid media (R, R+, Cos, Sch, CNS, YCFA, Chr, 2216E, BPN, BPN+B, BPN+L, BPN+B+L) and incubated at 37°C under anaerobic conditions for 48 hours. A 100 µL aliquot was streaked onto 5% sheep blood agar plates and incubated in an anaerobic workstation, with the process similarly repeated every three days for 30 days. The experimental workflow is shown in **Figure 1**.

#### 2.2.3 Single-colony isolation and identification

Colonies from aerobic and anaerobic conditions were selected from blood agar plates. DNA was extracted using a DNA mini-extraction kit (HiPure Fungal DNA Mini Kit, Catalog No: D3171-03, Magen Biotech Co., Guangzhou, China). PCR amplification of the 16S rRNA gene was performed using universal primers 27F/1429R (Frank et al., 2008). Amplification products (~1.4 kb) were verified by electrophoresis and submitted to Qingke Biotechnology for bidirectional Sanger sequencing (3730XI, AB, USA). Sequences were identified using BLAST (https://blast.ncbi.nlm.nih.gov/Blast.cgi).

### 2.3 Metagenomic analysis

#### 2.3.1 Microbial DNA extraction and library preparation

Platelet samples (5 mL) were centrifuged at 17,000×g for 10 minutes, and the supernatant was discarded. The pellet was resuspended in 100 µL sterile PBS. Microbial DNA was extracted using the DNA mini-extraction kit (HiPure Fungal DNA Mini Kit, Catalog No: D3171-03, Magen Biotech Co., Guangzhou, China). and used for library construction with the VAHTS^®^ Universal Plus DNA Library Prep Kit (Catalog No: ND617-02, Vazyme Biotech Co., Ltd., Nanjing, China) for Illumina. Libraries were quality-checked with a 2100 Bioanalyzer (G2939A, Agilent Technologies, USA), and sequenced using an Illumina NovaSeq 6000 platform (Novogene, China) to generate ~5 GB of raw data per sample.

#### 2.3.2 Bioinformatic analysis

Raw sequencing reads were quality-controlled using NGS QC Toolkit (Patel and Jain, 2012) (version 2.3.3). High-quality sequences were mapped and annotated against the Genome database (https://ftp.ncbi.nlm.nih.gov/genomes/) to determine taxonomic classifications. Microbial taxa in the platelet metagenome were identified using Kraken2 (Wood and Salzberg, 2014) (version 2.1.2), and taxonomic counts were corrected with Bracken (Lu et al., 2017) (version 2.9) to ensure accurate assignments at the lowest taxonomic levels. Data visualizations were performed using Python (version 3.11.7) with the Plotly Express library (version 5.9.0), Seaborn (version 0.12.2), and Matplotlib (version 3.8.0), as well as R software (version 4.1.3) with relevant visualization packages. Final adjustments to figure layouts were made using Adobe Acrobat Pro DC (version 2022). α-diversity included Observed richness (Sobs), Shannon H’, Simpson 1-D, Pielou’s evenness J, and Heip’s evenness. β-diversity was computed using Bray–Curtis (on relative abundances), Aitchison distances after CLR transform with a 1\times10^{-6} pseudocount, and Jaccard (presence/absence). Ordination used PCoA on each distance matrix (reporting variance explained by PC1/PC2), and hierarchical clustering used UPGMA on Bray–Curtis. Calculations were implemented in Python 3.11.7 (NumPy/Pandas/SciPy, Matplotlib/Seaborn/Plotly) and R 4.1.3.

### 2.4 Negative controls (culture-based and downstream metagenomics)

To monitor background and process-related contamination in this low-biomass setting, we included sterile PBS culture controls in parallel with platelet samples. For each culture run, 5 mL of sterile PBS was inoculated into the same aerobic and anaerobic fluid nutrient medium used for samples and incubated under identical conditions and schedule (temperature, atmosphere, agitation, transfer scheme, and total 30-day enrichment). Whenever sample cultures were subcultured to solid media, an aliquot of the corresponding control broth was plated onto Columbia blood agar to serve as a plating control. At day 30, the remaining volumes of control broths were subjected to DNA extraction, library preparation, and shotgun metagenomic sequencing alongside study samples, using the identical host-depletion, bioinformatic profiling, and reporting thresholds.

## 3 Results

### 3.1 Results of culturomics

**Figure 2:**
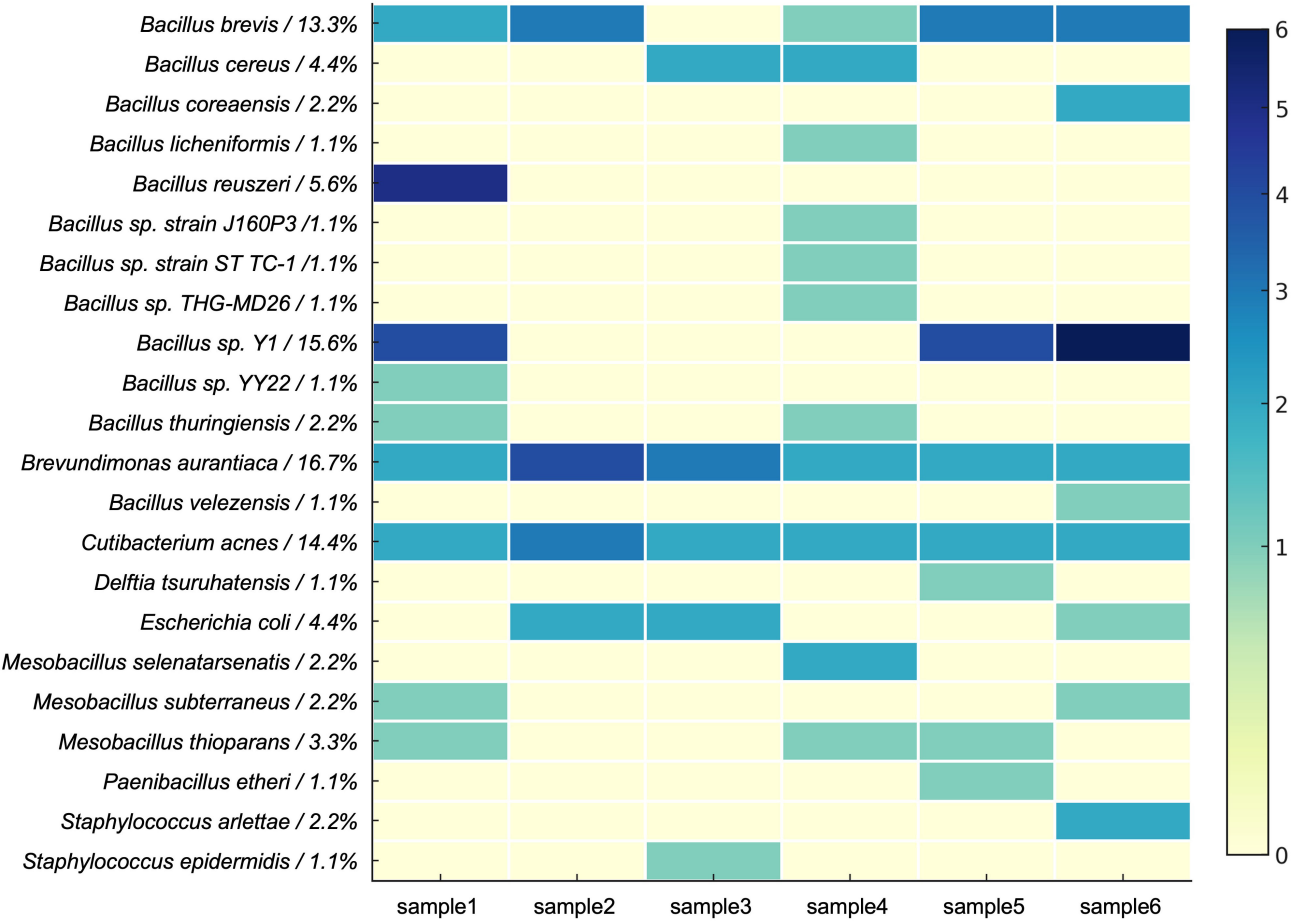
Bacterial species grown under microbial culturomics conditions from platelet samples.

Using liquid culture media plated on blood agar plates, single bacterial colonies were isolated. DNA was extracted from these single colonies, and PCR amplification was performed. Electrophoresis revealed an approximately 1.4 kb DNA band. Subsequent Sanger sequencing and BLAST analysis confirmed the isolation of a total of 90 bacterial strains from blood agar plates. The bacterial species identified from the six samples are shown in **Figure 2**. No colony growth was observed on control plates inoculated with sterile PBS, and nucleic acid extracted from the negative control samples at the end of the culture period showed “Too Low” concentrations as measured by Qubit, confirming the absence of contamination during the culture process.

**Figure 3:**
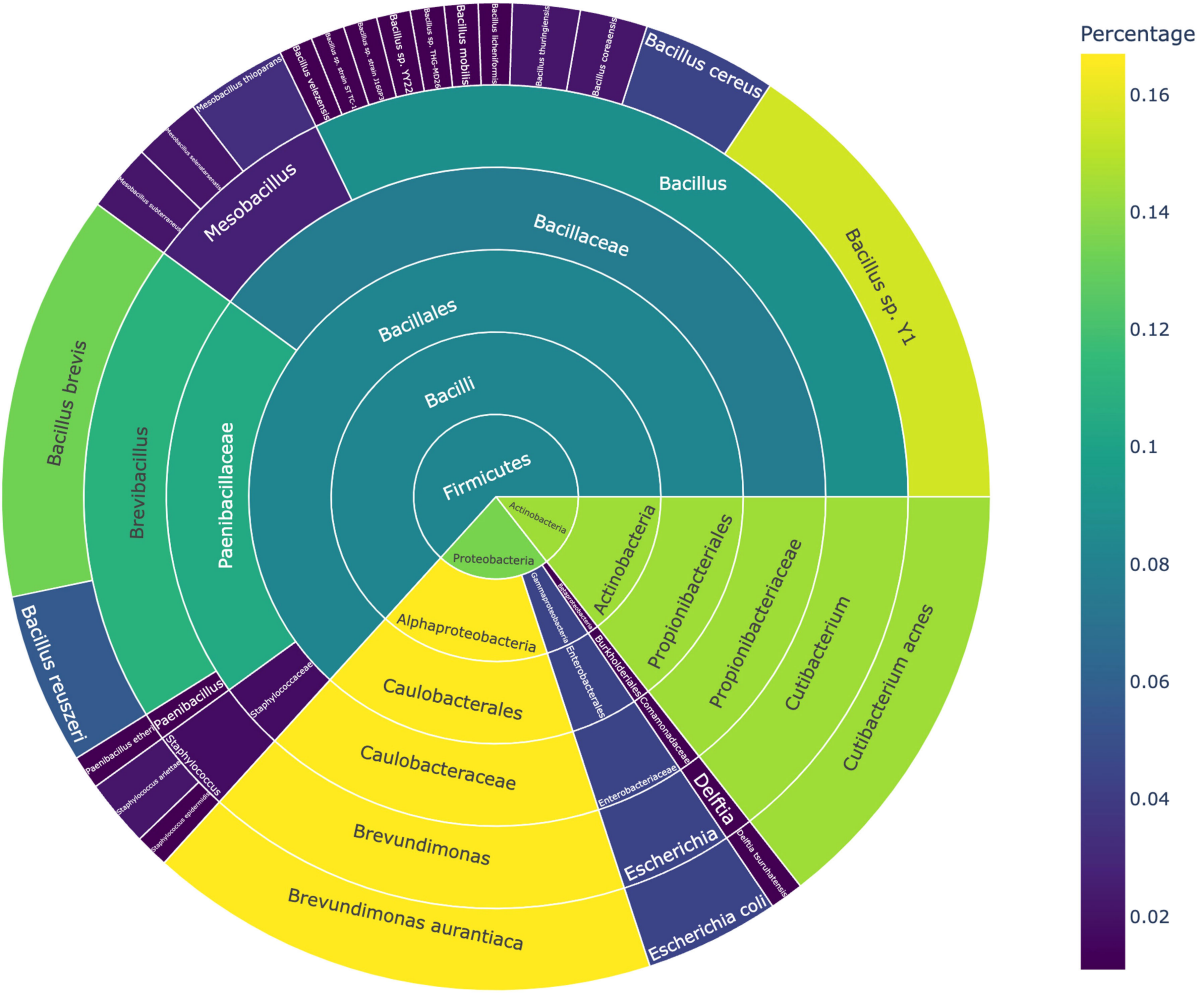
Classification and abundance distribution of bacteria grown under microbial culturomics conditions.

The isolated bacteria were classified into 3 phyla, 5 classes, 5 orders, 7 families, 9 genera, and 23 species (**Figure 3**). At the phylum level, Firmicutes dominated, with the highest number of strains isolated. The dominant genera included Bacillus and Brevibacillus, with specific species such as B. brevis and Bacillus sp. Y1 showing high relative abundances (13.3% and 15.6%, respectively). Among Actinobacteria, Cutibacterium acnes represented 14.4% of the total abundance. Proteobacteria was primarily represented by Brevundimonas aurantiaca, accounting for 16.7% of the relative abundance.

At the genus level, Brevundimonas, Brevibacillus, and Cutibacterium were the dominant genera, collectively accounting for 44.4% of the total abundance. Notably, Brevibacillus brevis, B. aurantiaca, and C. acnes were repeatedly isolated in at least five samples, suggesting that these species possess strong competitive advantages under the culture conditions.

### 3.2 Results of metagenomics

High-throughput sequencing using the Illumina NovaSeq 6000 platform generated 646.14 GB of raw data and 464.42 GB of clean data. The sequencing quality metrics were as follows: Q20 > 90%, Q30 > 85%, error rate = 0.03%, and an average GC content of 45.70%. Analysis of the metagenomic data from six platelet samples revealed the relative abundances of microbial taxa at the phylum, class, order, genus, and species levels.

#### 3.2.1 Relative abundance across phyla, class, orders, and families

**Figure 4:**
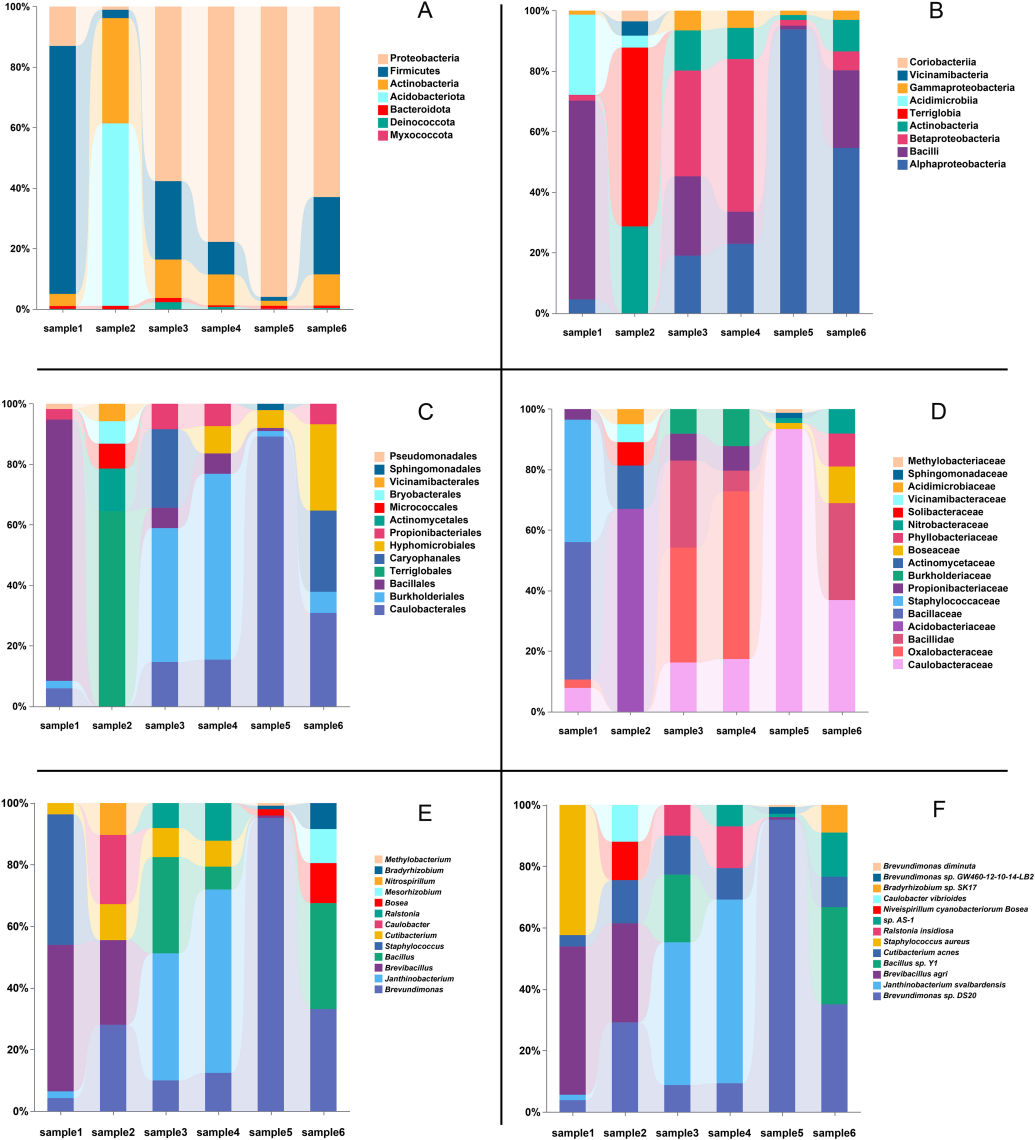
Relative abundance of microbial taxa across six different samples. **(A)** Relative abundance at the phylum level. **(B)** Relative abundance at the class level. **(C)** Relative abundance at the order level. **(D)** Relative abundance at the family level. **(E)** Relative abundance at the genus level. **(F)** Relative abundance at the species level. In each panel, the top five most abundant taxa within each sample are displayed, with the bars showing the percentage composition of each taxonomic group.

At the phylum level, the number of phyla identified in each sample ranged from 28 (samples 3 and 4) to 36 (sample 6) (**Figure 4A**). The most abundant phyla were Proteobacteria and Firmicutes, which dominated all samples. Specifically, Proteobacteria reached its highest average abundance in sample 5 (93.0%), while Firmicutes exhibited significantly higher abundance in sample 1 (82.0%). Additionally, Actinobacteria showed relatively high abundances in samples 2 (31.8%), 3 (10.9%), and 4 (9.6%).

At the class level (**Figure 4B**), Bacilli and Alphaproteobacteria were the most abundant classes, dominating all samples. Specifically, Bacilli reached its highest average abundance in sample 1 (86.3%), while Alphaproteobacteria exhibited significantly higher abundance in sample 5 (89.9%) and sample 6 (50.1%). Other classes, such as Betaproteobacteria and Actinobacteria, also showed notable presence in certain samples, with Betaproteobacteria reaching 46.9% in sample 4 and Actinobacteria peaking at 24.8% in sample 2. Terriglobia was predominantly detected in sample 2, where it contributed 51.1%, highlighting its unique abundance in this sample.

At the order level (**Figure 4C**), Bacillales emerged as one of the most prevalent bacterial orders, with a relative abundance exceeding 80% in Sample 1. Caulobacterales exhibited higher abundance in Samples 5 and 6, particularly in Sample 5, where it accounted for 81.6%. Additionally, other bacterial orders, such as Burkholderiales and Terriglobales, showed relatively high abundances in Samples 4 and 2, respectively.

At the family level (**Figure 4D**), Caulobacteraceae was identified as dominant bacterial families. Caulobacteraceae demonstrated notable abundance in Samples 5 and 6, reaching 81.6% and 24.4%, respectively. Bacillaceae accounted for 45.4% of the relative abundance in Sample 1. Other families, such as Staphylococcaceae and Oxalobacteraceae, exhibited higher abundances in Samples 1(40.4%) and 4(37.3%), respectively, indicating localized dominance of these bacterial communities in specific samples.

#### 3.2.2 Relative abundance at the genus level

At the genus level, variations in the dominant bacterial genera were observed across the samples. A total of 941, 1055, 849, 921, 1085, and 1039 genera were identified in samples 1 through 6, respectively (**Figure 4E**). Analysis of the dominant genera revealed that Brevundimonas was prevalent across all samples, with relatively high average abundance. Notably, its relative abundance reached 92.39% in samples 5, respectively, while ranging from 4.07% to 26.93% in the other samples, indicating its widespread presence. In contrast, Brevibacillus exhibited the highest relative abundance in samples 1 and 2, accounting for 45.35% and 21.79%, respectively. Janthinobacterium demonstrated significant dominance in samples 3 and 4, with relative abundances of 33.82% and 48.27%, respectively. Additionally, the known potential pathogen Staphylococcus showed a relatively high abundance of 40.40% in sample 1. Furthermore, low-abundance genera such as Ralstonia and Caulobacter were detected in certain samples, highlighting the diversity of the microbial communities present.

#### 3.2.3 Relative abundance at the species level

At the species level, the dominant bacterial species varied significantly among samples (**Figure 4F**). In samples 1 and 2, the most abundant species was B. agri, accounting for 46.03% and 25.0%, respectively. Notably, *S. aureus* also demonstrated a high relative abundance of 40.4% in sample 1. In samples 3 and 4, Janthinobacterium svalbardensis was the dominant species, with relative abundances of 37.7% and 48.1%, respectively. In samples 5 and 6, B. sp. DS20 was the most prevalent species, representing 92.7% and 29.2% of the relative abundance, respectively. Additionally, low-abundance species such as Ralstonia insidiosa and Delftia tsuruhatensis were detected, underscoring the capability of metagenomics to uncover microbial diversity.

### 3.3 Integrated analysis of culturomics and metagenomics

#### 3.3.1 Comparison of detected bacterial counts

**Figure 5:**
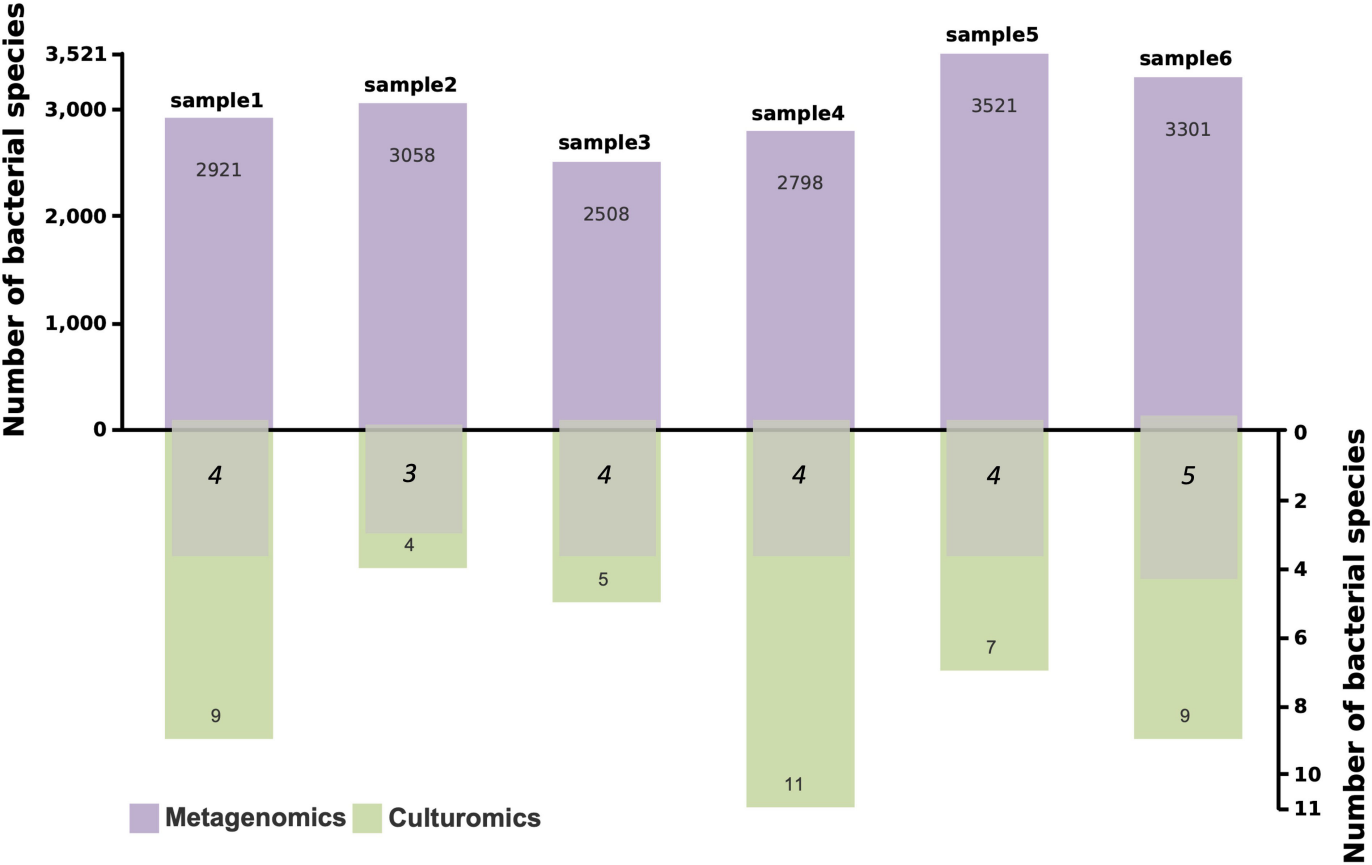
Bacterial species counts detected by metagenomics and culturomics, and overlapping bacterial species. The purple bars represent the number of bacterial species detected by metagenomics, the green bars represent the number detected by culturomics, and the overlapping numbers indicate bacterial species detected by both methods.

Metagenomics identified 2508 to 3521 bacterial species across six samples, with an average of 3018 species per sample, demonstrating its ability to capture the overall diversity of microbial communities. In contrast, culturomics detected a smaller number of bacterial species, ranging from 4 to 11 species per sample. Notably, the number of bacterial species detected by both methods ranged from 3 to 5 (**Figure 5**).

#### 3.3.2 Bacteria jointly confirmed by both methods

**Figure 6:**
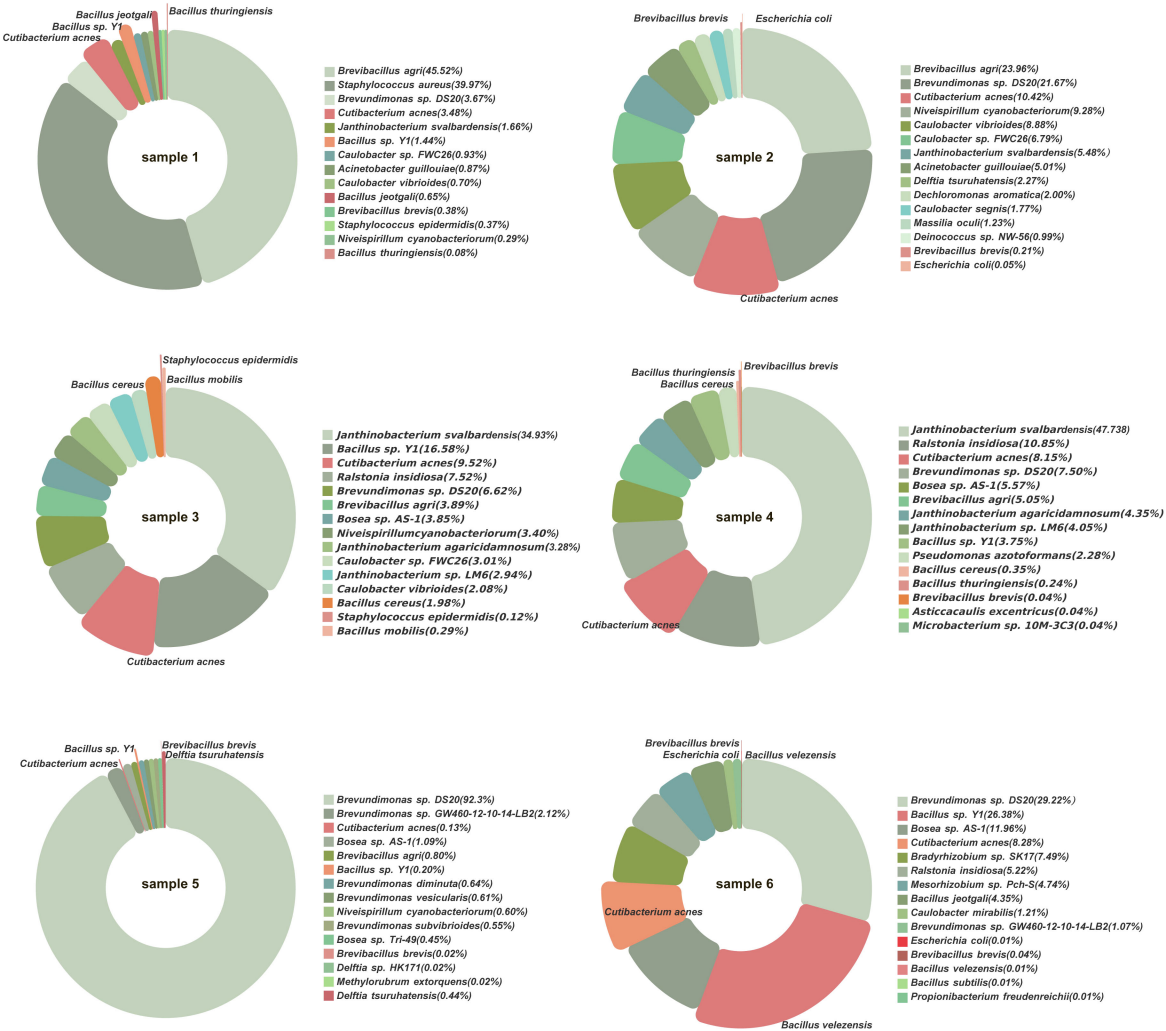
Bacterial species detected by both metagenomics and culturomics. The pie chart segments represent bacterial species with higher abundance in metagenomic sequencing, with the red-highlighted sections indicating cultivable bacteria successfully grown in culturomics.

A total of 10 bacterial species were identified by both culturomics and metagenomics (**Figure 6**). These included B. sp. Y1, B. thuringiensis, C. acnes, B. brevis, Escherichia coli, S. epidermidis, B. cereus, B. mobilis, D. tsuruhatensis, and B. velezensis. Among these, species such as C. acnes and B. sp. Y1 exhibited relatively high abundances in multiple samples, indicating strong adaptability and competitiveness under culture conditions.

Additionally, some bacteria with low relative abundances in metagenomics, such as D. tsuruhatensis and B. velezensis, were successfully isolated through culturomics, emphasizing the complementary nature of the two methods. Culturomics provides direct experimental evidence for the viability of these bacteria and their potential roles in the microbial community.

### 3.4 Diversity landscape of platelet samples

Across samples, within-sample (α) diversity showed high richness but variable evenness (Observed richness 849–1083 genera; Shannon H′ 1.23–3.63; Simpson 1−D 0.307–0.925; Pielou’s J 0.176–0.521). Sample 5 exhibited a “high-richness/low-evenness” pattern (Shannon H′=1.23; Pielou’s J = 0.176), indicating dominance by one or a few taxa despite many detected genera, whereas samples 2–3 showed both high richness and high evenness (Shannon H′ ≥ 3.37; Simpson 1−D ≥ 0.892). Between-sample (β) diversity was substantial: mean pairwise dissimilarity was 0.73 (Bray–Curtis; range 0.26–0.97), 69.25 (Aitchison/CLR Euclidean; 42.39–101.07), and 0.24 (Jaccard; 0.14–0.32). PCoA based on Bray–Curtis explained 53.5% and 34.3% of variance on PC1 and PC2, respectively (Aitchison: 59.3%/21.6%; Jaccard: 46.7%/26.3%), yielding dispersed ordinations without tight clustering—consistent with heterogeneous community structure across donors. Distance matrices were moderately correlated for Bray versus Aitchison (Spearman ρ=0.67, p≈0.033) and weaker for comparisons involving Jaccard (ρ≤0.58), reflecting the difference between abundance-based and presence/absence metrics.

### 3.5 Negative controls

No colony growth was observed on blood agar for the PBS culture control during the 30-day enrichment. The PBS control processed in parallel failed library qualification: library qPCR was negative (Ct undetermined), consistent with insufficient yield/library-preparation failure. Accordingly, this control was considered non-informative for metagenomic contamination assessment and was excluded from sequencing and downstream taxonomic analyses.

## 4 Discussion

### 4.1 Potential threats of bacterial contamination in platelets

Unlike refrigerated red blood cells or frozen plasma, platelet products require room-temperature storage under continuous agitation to maintain functionality, creating favorable environments for bacterial proliferation. The elevated risk of bacterial contamination in platelet products has been well-documented in hemovigilance systems from Europe and North America, where routine bacterial screening is now standardized to mitigate transfusion-associated risks (Levy et al., 2018).In contrast, China has yet to implement systematic bacterial culturing screening protocols for platelet products (National Health Commission of the People’s Republic of China, 2019). To mitigate adverse reactions during platelet transfusion, many physicians in China administer hydrocortisone or dexamethasone to patients either before or during the transfusion to prevent fever and other symptoms (Dazhang et al., 2002). While this practice may reduce reported rate of transfusion-related bacterial infections, and could potentially lead to underdiagnosis and delayed therapeutic interventions in clinical practice (Delaney et al., 2016).

Our data demonstrate a diverse bacterial consortium within platelet samples, raising serious concerns about their potential impact on Chinese blood recipients, particularly those immunocompromised populations. Culturomic analyses confirmed the presence of S. epidermidis (Becker et al., 2014; Vuong and Otto, 2002), E. coli (Russo and Johnson, 2003; Pitout and Laupland, 2008), and *S. aureus* (Chambers and Deleo, 2009; Tong et al., 2015), as well as C. acnes (Achermann et al., 2014; Vollmer et al., 2014) detected via metagenomics, were all well-documented pathogens associated with bloodstream infections (BSIs). Notably, emerging opportunistic pathogens such as D. tsuruhatensis (linked to biofilm-mediated catheter-associated BSIs (Ranc et al., 2018).) and Brevundimonas spp. (e.g., B. vesicularis and B. diminuta, implicated in immunocompromised-host infections (Ryan and Pembroke, 2018)) were also detected. Other bacteria identified, including R. insidiosa (persistence in medical equipment (Ruggieri et al., 2024; Alnimr, 2023) and carbapenem-resistant (Fang et al., 2019)), B. cereus (associated with hematologic malignancy-related BSIs (Bottone, 2010; Gaur et al., 2001)) are generally considered environmental microorganisms but may pose infection risks under specific conditions. Some bacteria detected in this study, such as B. sp. AS-1 and B. sp. Tri-49 from the genus Bosea, B. brevis, B. velezensis, B. coreaensis, B. licheniformis, and B. thuringiensis from the genus Bacillus, and C. vibrioides from the genus Caulobacter, currently lack direct evidence of pathogenicity, however, phylogenetically related strains [e.g., B. thiooxidans in catheter infections (Skipper et al., 2020)] and clinical reports suggest they may occasionally act as opportunistic pathogens (Justesen et al., 2007; Moore and Gitai, 2020).

These findings emphasize the vulnerability of high-risk cohorts—immunosuppressed patients (e.g., post-transplant or chemotherapy recipients), neonates, and the elderly—to severe outcomes from platelet-transmitted BSIs. Comprehensive risk stratification and vigilant monitoring are imperative to mitigate morbidity in these populations.

### 4.2 Co-existing’ bacteria in platelets: interpreting low-abundance microbial signals

The identification of diverse microbial communities in platelet products not only underscores contamination risks but also challenges the historical paradigm of blood sterility. Over the past two decades, advancements in microscopy and bioinformatics technologies have significantly advanced our understanding of microbial presence in the bloodstream (Whittle et al., 2018; Li et al., 2018; Bhattacharyya et al., 2017; Paisse et al., 2016; Potgieter et al., 2015; Dinakaran et al., 2014; Rajendhran et al., 2013; Nikkari et al., 2001). For instance, Potgieter et al. visualized bacteria associated with blood cells in various blood products using electron microscopy (Potgieter et al., 2015), while Damgaard et al. isolated viable bacteria from 62% of healthy donor samples (Damgaard et al., 2015). Currently, evidence regarding microorganisms detected in human blood is not consistent. A large-scale sequencing study of 9,770 healthy individuals found no shared, stable core blood microbiome, but did confirm the detection of bacteria in 16% of individuals, with nearly 5% harboring the same bacterial species (Tan et al., 2023). In healthy individuals, microorganisms detected in blood can be interpreted along two clinically distinct patterns: (i) bona fide pathogens may transiently enter the bloodstream but are rapidly cleared by innate and adaptive immunity, leaving no sustained clinical syndrome; this phenomenon is well documented for viridans group streptococci and other mucosa-derived taxa, with bacteremia typically peaking within minutes and declining thereafter (Martins et al., 2024; Wilson et al., 2021); (ii) some studies have reported viable bacteria at very low levels in freshly drawn blood from self-reported healthy donors (Damgaard et al., 2015)—potentially consistent with low-metabolic or VBNC (viable but non-culturable) -like states (Oliver, 2010). The existence and biological significance of these so-called ‘co-existing’ bacteria in blood remain controversial and have therefore attracted widespread attention (Païssé et al., 2016; Damgaard et al., 2015, D’aquila et al., 2021).

In this context, our inference about a putative endogenous co-existing bacteria is grounded in a conservative, clinically anchored interpretation of low-abundance signals. First, transient translocation of bona fide commensals and pathobionts can occur and is typically self-limited in immunocompetent hosts (Lehtiniemi et al., 2005; Whittle et al., 2018). This aligns with our findings of species linked to these body sites, including common skin commensals(C. acnes, S. epidermidis, B. velezensis, B. cereus, B. licheniformis, and *S. aureus* (Fournière et al., 2020; Dréno et al., 2020)), gut microbes (B. thuringiensis and E. coli (Brenchley and Douek, 2012) (Di Marco Barros et al., 2022)), and oral commensals (Brevundimonas, Paenibacillus, Delftia, and Staphylococcus (Sarkar et al., 2017; Nasidze et al., 2009; Yamashita and Takeshita, 2017; Horliana et al., 2014)). Once in the bloodstream, these bacteria may enter a dormant state, consistent with the concept of “viable but non-culturable” (VBNC) bacteria, which maintain metabolically active but cease division, regaining activity and proliferation under favorable condition (Wagley et al., 2021; Mu et al., 2021; Kell and Pretorius, 2015; Potgieter et al., 2015). However, DNA-only metagenomics cannot by itself adjudicate viability or a viable-but-non-culturable (VBNC) state *in vivo*, and such claims require orthogonal evidence (e.g., culture resuscitation, rRNA/metatranscriptomic signals, or chemical exclusion of dead-cell DNA). Accordingly, we regard ‘healthy bacteremia’ as a speculative concept and interpret our low-abundance findings with caution.

The possible existence of blood co-existing bacteria has raised questions about their functional roles in the human bloodstream. Certain blood commensal bacteria, such as C. acnes and S. epidermidis, can evade immune detection and establish long-term colonization through biofilm formation, or finely tuned metabolic and virulence adaptations (Becker et al., 2014) (Achermann et al., 2014) (Lê-Bury et al., 2024), highlighting their potential adaptive mechanisms for survival in the bloodstream and warranting further investigation. In interpreting low-abundance blood detections, host context is pivotal. Recent case evidence shows that immunocompromised patients may harbor invasive, hyper-aerotolerant Campylobacter jejuni capable of causing bacteremia, underscoring that barrier-site organisms can exhibit atypical, blood-intruding phenotypes in specific hosts (Zeng et al., 2025). Consistent with prior work on hyper-aerotolerant Campylobacter, enhanced oxygen tolerance may facilitate survival outside microaerophilic niches and increase the likelihood of extraintestinal invasion (Oh et al., 2019). Interestingly, healthy individuals with a lower abundance of Proteobacteria and a higher abundance of Actinobacteria in their blood are reportedly at a reduced risk of type 2 diabetes (Amar et al., 2011), while bacterial 16S rRNA gene concentrations tend to increase with age (Yun et al., 2019),though the precise roles of specific bacteria in health and disease progression remain unclear and warrant further investigation.

### 4.3 Integrated methodological and clinical implications

Tan et al. (2023) proposed that the blood microbiome is not a stable, resident community, but rather represents a sporadic and transient presence of microorganisms originating from other body sites (Tan et al., 2023). If a core microbial community existed in the bloodstream, targeted purification strategies could potentially be employed in clinical practice to ensure transfusion safety. However, the existence of a heterogeneous and fluctuating microbiome presents a greater challenge to transfusion medicine. Our 30-day extended culturomic approach revealed the microbial diversity within platelet concentrates. In natural environments, certain bacteria exist in oligotrophic states (Ho et al., 2017). While high nutrient concentrations in culture media may have little effect on fast-growing and resilient bacteria, they can harm slow-growing, less adaptive bacteria, even leading to their death (Bartelme et al., 2020). We speculate that the 30-day extended culture period gradually reduced nutrient levels in the media, creating conditions more favorable for the growth and isolation of oligotrophic bacteria (Zhang et al., 2021). In our unpublished study, we explored the temporal dynamics of microbial communities by analyzing bacterial abundance and composition at specific time points (Days 3, 5, and 10). Fast-growing aerobes (e.g., R. insidiosa) peaked by Day 5, whereas anaerobes (C. acnes) thrived throughout storage, reflecting their metabolic adaptations. Additionally, P. spp. and S. maltophilia exhibited significant changes in abundance at different time points, suggesting dynamic shifts in their growth patterns during storage. Such temporal profiling identifies high-risk contaminants, advocating for early screening to preempt pathogen proliferation.

This study integrated and compared culturomics and metagenomics to emphasize their complementary strengths in detecting bacterial contamination in platelet samples. Culturomics isolated viable strains (e.g., B. aurantiaca) for phenotypic analyses, such as antibiotic susceptibility and metabolic profiling, which are critical for understanding pathogenic potential and designing interventions. However, it favors fast-growing or high-abundance microorganisms (Lagier et al., 2015; Browne et al., 2016), often overlooking low-abundance or slow-growing species requiring specific growth conditions. In contrast, metagenomics provided a culture-independent approach, detecting unculturable and rare taxa (e.g., B. sp. DS20) by capturing the genetic diversity and functional potential of microbial communities. This method is particularly suitable for detecting bacteria that are difficult to culture under laboratory conditions, such as obligate intracellular pathogens or extremophiles. Nonetheless, metagenomics cannot confirm viability and faces challenges like reduced sensitivity for low-abundance microbes, taxonomic resolution limitations, and depth biases (Boolchandani et al., 2019). Future Integration of long-read sequencing technologies (e.g., nanopore sequencing (Ying et al., 2022)) could mitigate these gaps, enhancing detection of rare or slow-growing pathogens.

### 4.4 Contamination risk and interpretive safeguards in a low-biomass setting

Sequence-based surveys of blood or platelet products are intrinsically vulnerable to reagent-borne DNA (“kitome”), environmental/handling carry-over, well-to-well exchange, and index misassignment, all of which can distort community profiles at low biomass. Prior work has shown that contaminant DNA is ubiquitous in extraction kits and varies by kit/batch, with disproportionate impact on low-biomass shotgun and amplicon data; current best-practice recommendations emphasize rigorous negative controls and conservative interpretation (Salter et al., 2014; Eisenhofer et al., 2019).

What we did in this study. (i) At collection, we followed the blood center’s industry-standard antisepsis and sterility procedures (alcoholic chlorhexidine prep with specified scrub/dry times, first-diversion of the initial 20–30 mL, sterile-port access, no-touch technique). In the laboratory, all aliquots were handled in a Class II biosafety cabinet using dedicated, pre-cleaned instruments and aerosol-resistant tips to limit operator-derived contamination. (ii) Each processing batch included a PBS-only culture control that underwent the same 30-day enrichment and day-30 metagenomic workflow as study samples; these blanks were used to monitor process background.

Known risks we monitored but did not fully quantify. We recognize risks from well-to-well exchange during plate-based extractions and index misassignment (“index hopping”) during multiplexed sequencing. These phenomena can spuriously spread reads across samples and inflate apparent diversity. For low-biomass datasets, we recommend adopting conservative interpretive rules—namely: treat low-abundance taxa plausibly linked to skin or reagents with particular caution; prioritize signals only when (a) they exceed blank maxima, (b) are reproducible across assays/media, or (c) are concordant with clinical/biological context (Eisenhofer et al., 2019; Costello et al., 2018). Although our run design avoided mixing obvious high-biomass libraries with platelet samples, and blanks were interleaved to flag cross-talk, the present dataset was not powered to estimate these effects precisely; future runs will adopt unique dual indexing and tighter plate layouts to further suppress misassignment and well-to-well transfer (Davis et al., 2018).

## 5 Conclusion

The integration of culturomics and metagenomics offers a transformative framework for transfusion safety, bridging the detection of viable pathogens and microbial diversity. We believe that the combination of these approaches will not only help the more vulnerable recipients, but also demonstrate a new way to prioritize elucidating the functional roles of blood-associated microbiota and optimizing platelet storage protocols, as to providing novel technical support to enhance blood safety.

## 6 Limitations

The principal limitation of this study is the modest sample size, which diminishes the ability to detect and precisely estimate low-abundance/rare bacteria and restricts comparisons across collection batches, processing procedures, or donor characteristics, thereby limiting external generalizability. In addition, the absence of technical replication and the limited number of biological replicates further reduce precision for low-abundance taxa and preclude robust estimation of batch effects. As a low-biomass investigation, residual process/reagent or handling background cannot be completely excluded; accordingly, we interpret low-abundance skin commensals cautiously, prioritizing signals with reproducibility across assays, higher quantitative burden, or clinical correlation. To better attribute signals to blood-derived bacteria and evaluate potential venipuncture contamination, future work will include paired skin-surface swabs collected adjacent to the venipuncture site at the time of whole-blood sampling, using a standardized saline-moistened swabbing protocol (press and swab at ~45°, followed by a perpendicular pass, repeated cycles), with swab tips pooled into sterile cryovials as paired skin controls. This study was designed to establish the feasibility and complementarity of combining long-term culturomics with metagenomic sequencing for bacterial detection in platelet products; accordingly, our findings are best interpreted as hypothesis-generating and methodological signals rather than definitive population-level inferences. Future studies should replicate and extend these results in larger, multi-center cohorts under a pre-specified analysis plan, incorporating unit-level and technical replication and comprehensive contamination controls.

## Data Availability

Publicly available datasets were analyzed in this study. This data can be found here: The raw data of metagenomes have been submitted to the China National GeneBank DataBase (https://db.cngb.org/) with the accession number CNP0006958.
